# A System of Surgery

**Published:** 1848-03

**Authors:** 


ART. IV.—*A System of Surgery.* By J. M. CHELIUS. Translated by John F. South. (*Review continued from page* 536.)

*Third Division. Unnatural Coherence. Of fingers and toes, of joint-ends, of bones, of nostrils, of the tongue.*

"The accidents which may occur after separating the tongue in the above cases are bleeding, and when the tongue has been divided to a great extent there is danger of suffocation. Attempts to stop bleeding of the wounded ranine artery must be made with little bundles of lint, moistened in styptics, Theden's arquebusade, or a solution of alum, which should be pressed against it with the finger ; or the actual cautery may be applied to the bleeding part, which in all cases is preferable to the compressors of Petit, Jourdain, and Lampe. Bleeding may also be produced by the child sucking his tongue ; in which case the blood will be swallowed. Care must, therefore, be taken for the first few days, that when he awake he be laid on his breast."

" Widening of the mouth by cutting at both corners, as formerly advised is useless, as the cut always again unites, and the deformity is worse. The treatment must vary according to the several degrees of narrowing and destruction of the lips."

We have succeeded perfectly in several instances in enlarging the mouth where it was preternaturally narrowed, by cutting to the required extent at the angles, and then attaching the mucous membrane to the skin by several delicate sutures. In this way, the mouth may be enlarged to any extent. We do not claim this practice as original with us, but we are not now prepared to say by whom it was first suggested.

*Stricture of the œsophagus.*—" The *prognosis*, in stricture of the *œsophagus*, depends on the kind and seat of that affection. The simple, membranous stricture admits a favorable *prognosis*, and may be completely got rid of by proper treatment. Callous and scirrhous stricture rarely allows any check to its developement, and, when one existing, its alleviation is scarcely possible, and still less its cure. Spasmodic stricture may mostly be got rid of by proper treatment; the disease however often recurs, is frequently very stubborn, and may run into organic stricture. In swellings which compress the *œsophagus*, the *prognosis* depends on their nature and seat."

Speaking of the introduction of a sound for the purpose of dilating a membranous stricture, Mr. South makes the following judicious remarks :—

"The introduction of the sound through the nostril is improper, much more painful to the patient, and, if it be thick, impossible; by this mode also the iustrument more frequently slips into the windpipe, than in passing it by the mouth. This is proved by the violent disposition to cough, by the expulsion of the air with the sound, and the impossibility of speaking; it is right, however, to observe that frequently the disposition to cough, although the sound be thrust into the windpipe, is not so great; and that by the choking, when the sound is in the *œsophagus*, the air may be forced up from the stomach with a hissing noise."

In relation to Chelius's advice to protrude the tongue when entering the sound, Mr. South also observes :—

"The practice here recommended, of protruding the tongue, preparatory to passing the sound or bougie, and which, I believe, was first recommended by Home, is very objectionable, as in proportion must the *epiglottis* be raised, and greater facility for the entrance of the instrument into the windpipe afforded. Moreover, it is quite needless, as the passage into the beginning of the *œsophagus* is rendered easy by placing two fingers of the left hand upon the tongue, and pressing it into the bottom of the mouth, which readily exposes the whole cavity of the *pharynx*."

*Stricture, &c., of the rectum*, is a long and valuable chapter; *phimosis, paraphimosis and stricture of the urethra*, succeed: the latter is so replete with excellent suggestions, that we should be glad to copy it entire; indeed, in reading this volume for the purpose of presenting a condensed review of it, we have again and again stopped and looked forward to see whether any so valuable matter could be found in the succeeding pages, and again each time we have turned back and been compelled to admit that it is in every page replete with excellent advice. We cannot give preference where all is equally good, and no choice remains but to copy the whole or to select at random.

*Closure and narrowing of the vagina and womb* completes this *division*.

*Fourth Division. Diseases dependent upon the presence of foreign bodies.* This division is arranged by our author in the following manner :—

I. Foreign Bodies brought into our organism from without,
1. Into the nostrils.
2. ——— mouth.
3. ——— œsophagus and intestinal canal.
4. ——— air tube.

II. Unnatural collections of natural products.
A. In their natural cavities and receptacles (retentions.)
1. Ranula.
2. Retention of urine.
3. ————— the fœtus in the womb or in the belly (Cæsarean operation, division of the *pubes*, Gastrotomy.)

B. External to their proper cavities and receptacles (extravasations.)
1. Blood-swellings in newly-born children.
2. Hæmatocle.
3. Extravasation of blood in joints.

III. Collections of diseased products.
1. Lymph swellings.
2. Dropsy of the mucous bags.
3. ———— joints.
4. ———— the chest and *empyema.*
5. ———— the head and *spina bifida.*
6. Collections of pus in the breast-bone.
7. Dropsy of the *pericardium.*
8. ———— belly.
9. ———— ovaries.
10. Hydrocele.

IV. Formation of stony concretions.

*Internal use of stone solvents.*—"For stones consisting of uric acid, alkalies, for those of phosphates, the use of acids have been recommended, with corresponding dietetic treatment. In regard to acids, it must also be added, that they operate only against the phosphatic *diathesis*, and probably have no effect in dissolving stones, except by injection.

"Many empirical remedies consist principally of alkalies, as Stephens's remedy, and others. Some, as so many of the vegetable remedies, operate only by alleviation, through the large quantity of drink combined with their use. Many mineral waters operate both ways.

"The efficacy of these remedies has been too highly valued by many practitioners, and by others too much decried. If we cannot expect by the use of these remedies to dissolve large stones, yet, however, their increase may be prevented, the symptoms caused by the stone diminished, and small stones perhaps got rid of. Under circumstances which forbid the removal of a large stone by operation, or after the performance of an operation to get rid of the *diathesis* producing stone, their employment is very advantageous."

The following remarks on the solution of calculi, by injections into the bladder, are contained in the notes :—

"The most recent experiments made on this subject are those of Pelouze, from which it appears—*First*, that the effect of different agents upon urinary stones is less upon the substances of which they consist, than upon the animal matter. The operation proceeds very slowly, even out of the bladder. *Second*, that by drinks and baths a cure is scarcely ever effected. *Third*, that the result of injections, although they act more powerfully, is problematical, and the danger of inflammation is not counterbalanced, as in lithotrity, by a quick destruction of the stone. *Fourth*, that although the combination of lithotrity with injections increases the probability of success, yet it is most advisable to proceed with lithotrity.

"Prout observes :—'When the very weak state of the solvent that can be injected into the bladder is taken into account, the consequent length of the time necessary for continuing the experiment, and above all the refractory nature of certain *calculi*, I confess I am very much disposed to doubt if any solvent at present known can, in the great majority of instances, be ever so administered as to produce the desired effect; and this, I believe, is the general opinion on the subject.'

"'It has also been observed by chemists,' says Brodie, 'that lithic acid admits of being dissolved by a strong solution of pure or caustic alkali. It has been also observed that *calculi* composed of the phosphates are acted on by the mineral acids, and it may not unreasonably be entertained as a question, how far those changes, which take place out of the body, may be produced while the *calculus* is still in the bladder of a living person. * * * I fear those who have expected by these methods to relieve patients of lithic acid *calculi*, have much overrated the effects of alkaline *lixivia* on them. The fact is, that although alkalies certainly are capable of acting on this kind of *calculus*, their action, except when employed in a very concentrated form, is so inconsiderable, as to amount almost to nothing. Neither the stomach nor the bladder is capable of bearing the quantity of alkali which is necessary to the production of the desired effect; and even if they were, it would be impossible to maintain so constant a supply of the alkali as would be necessary to the destruction of a *calculus* of even moderate dimensions. Mr. Brande, moreover, has observed, that the carbonate of potass and soda have no action on lithic acid; that they are incapable of dissolving it; and that if the pure alkili be taken by the mouth, it never reaches the bladder in this state, but only in that of a carbonate; and here then is an insuperable objection to all attempts to dissolve lithic (uric) acid *calculi* by means of alkalies taken into the stomach. When there is a lithic (uric) acid *calculus* in the bladder, and the lithic acid *diathesis* prevails in the system, the first effect of alkalies taken into the stomach is to render the urine neutral; thus preventing the farther increase of the *calculus*. So far, then, alkalies are useful. But if they are administered in still larger quantity, so as to render the urine alkaline, the phosphates begin to be deposited. The *calculus* then continues to grow even more rapidly than before; but its composition is altered, and layers of the triple phosphate are deposited on the lithic acid nucleus. Such is the view of the subject taken by Mr. Brande.'

"Brodie shows the fallacy of the statements in reference to the presumed solution of stone, by observing that the fragments occasionally passing, are to be referred to fracture of the stone from mechanical causes, as already mentioned; or that the supposed fragments are in reality new formations, and the result of the medicines employed. As to the cessation of the symptoms, it is no proof the solution of the stone, as by the use of medicine, a fresh coating may be given to it of a less irritating character, and the stone still exist, as in the case of Admiral Douglas, mentioned by Astley Cooper.

"Brodie, however. considers that 'the mineral acids undoubtedly exercise a greater chemical action on *calculi* composed of the phosphates than alkalies do on those which are composed of lithic (uric) acid. * * * I found that where the mucous membrane of the bladder was not inflamed at all, or inflamed only in a slight degree, the proportion of nitric acid might be increased to two minims or two minims and a half of the concentrated acid to an ounce of distilled water, without any ill consequence or even inconvenience arising from it. I next endeavored to ascertain to what extent a solution of this strength was capable of acting on a *calcuus* of the mixed phosphates. The change produced was sufficiently obvious, especially when the solution was made to pass over the *calculus* in a stream for a considerable time. It gradually diminished in size, and at last began to be broken down into minute fragments.' For this purpose he at first used a gold catheter with a double channel, through which a constant current was kept up; but afterwards found an elastic gum bottle with a stop-cock, and elastic gum tube attached to it. He first washed out the bladder with distilled water to get rid of the *mucus* lodged in it, and then injected the solution of nitric acid very slowly, using the same liquid over and over again several times. The liquid was afterwards tested with a highly concentrated solution of pure ammonia, and it was found that if the ammonia was added in sufficient, but not too large quantity, the phosphates were precipitated in abundance. Hence he concludes, '*first*, that a *calculus*, composed externally of the phosphates, may be acted on by this injection, so as to become gradually reduced in size, while it is still in the bladder of a living person; *second*, that there is reason to believe that small *calculi*, composed throughout of the mixed phosphates, such as one met with in some cases of diseased prostate gland and bladder, are capable of being entirely dissolved under this mode of treatment, and that it is probable that it may therefore be applied with advantage to some of these cases in which, from the contracted state of the bladder, or from other circumstances, the extraction of such *calculi* by means of the urethra-forceps, cannot be accomplished.'"

Attempts have also been made by several persons to dissolve calculi by means of the galvanic pile. The proposal was first made by Gruithuisen; Prevost and Dumas have experimented upon animals, and it is said that an American, named Hull, made the attempt upon a man in London in 1844: with what success is not known.

*Fifth Division. Diseases which consist in the degeneration of organic parts, or in the production of new structures,* including many varieties of tumors, polypi cancers, &c. &c.

"Cancer of the womb, like cancer in general, exhibits many varieties in its progress; in persons with dense fibre, it is rather the progressive ulceration of scirrhous parts, but in pasty persons it is mostly accompanied with fungous growths and very copious bleedings.

"The diagnosis is in general easy, and the more so as the practitioner is usually only first consulted when the disease has made some progress.

"Those diseased conditions, which at first have some resemblance to cancer of the womb, but are easily distinguishable, are chronic inflammation and benignant hardening, steatomatous (fibrous degeneration,) eversion of the womb, polyp, and medullary fungus.

"The 'Cauliflower Excrescence from the Os Uteri,' as it is called by Dr. Clark, is a form of disease by some regarded as truly cancerous, and by others as a morbid tissue, not necessarily of a malignant or carcinomatous nature. Upon this point, Simpson observes:—'A number of circumstances appear to me to show, that, in reference to, at least, the first stage of cauliflower excrescence, the opinion of these latter authors is probably correct. The occurrence of the disease in some cases as early as the twentieth year of life; its occasional shrinking, and almost total disappearance upon the application of a ligature, or after death; the frequent slowness of its general progress during life; the apparent absence of diseased deposits in the neighboring tissues and parts upon the dead body; and above all the alleged restriction, and even complete removal of the tumor in one or two instances, by the use of astringent applications and other simple means, form so many circumstances strongly pointing to the opinion, that in the earlier part of its progress, the tumor cannot be regarded as of a carcinomatous character. Has it any analogy in its pathological nature and origin—as it certainly has in its physical characters—with the soft warts and condyiomata that sometimes form on the mucous membrane of the vulva and entrance of the vagina? Those warts and condylomata have the same tendency to degeneration after their imperfect removal, and present to us a striking exception to the general pathological law of the local reproduction of a morbid growth being a sign of its malignancy. But whatever view we may take of the primary nature of the cauliflower excrescence of the cervix uteri, we have sufficient evidence for believing either that this disease has been often confounded with carcinomatous or medullary fungus from the cervix uteri, from the want of adequate diagnostic marks to distingish them; or that, though non-malignant in its commencement, the cauliflower excrescence may, like some other local benign growths, become the seat of carcinomatous deposit and malignant action, during its progress.' May the degree of mobility of the cervix uteri serve in any case as a source of diagnosis? 'The tendency of cancer,' as observed by Muller, 'is to interfere with the natural structure of surrounding parts, while these formations which are of a benignant nature, leave the neighboring healthy tissues unaltered.' In carcinomata of the cervix uteri, we thus generally find, even at a pretty early stage of the disease, that the organ has become more *fixed* and immovable than natural, in consequence of the morbid deposit affecting both the structure of the neck of the organ and the contiguous surrounding tissues. Does the reverse of this hold good with regard to cauliflower excrescence of the cervix uteri?"

*Sixth Division. Loss of organic parts.* Declaring the various modes of restoring lost or mutilated parts, including artificial or mechanical substitutes for limbs, &c.

*Seventh Division. Superfluity of organic parts*, such as supernumerary fingers, toes, teeth and double nose!

*Eighth Divisisn. Elementary management of surgical operations.* This is the final division, and considers all the operations in minor surgery such as bleeding, cupping, application of issues, introduction of setosn, also the major operations of amputation, pesection, &c.

On the whole, we rise from the perusal of this work with the conviction that if, in its omission of several important branches of surgery, it cannot be called a complete book upon surgery, yet it is one of the most learned and practical writings extant. It must at once take a place, wherever it is known, among the standard surgical authorities. The reader will however not fail to notice certain German-isms, or what we might style, medical Carlyle-isms, such as the almost constant use of the "radicals" jaw-bone, collar-bone, blade-bone, upper-arm-bone, cubit-bone, spoke-bone, mid-hand-bone, shin-bone, splint-bone, joint-ends, air-tube, tongue-string, &c., to which Mr. South has added not a few, such gruted cockney-isms, as "come to a stand-still," "hinder-end" and "front-end," with the scarcely less elegant terms "gut" and "belly," and a variety of other select phrases which since the days of Abernethy have been considered obsolete. Had an American written in this style of vulgar affectation, it would have furnished material for a volume of satire to some narrow brimmed Englishman. But a surgeon of St. Thomas may write and speak as he shall choose, either in the defence of his neighbors at Billingsgate, or of his friends Fagin and Likes at St. Giles, and no one dare say it is not in good taste.

